# Supplementary material for: Common Inflammation-Related Candidate Gene Variants and Acute Kidney Injury in 2647 Critically Ill Finnish Patients
Source: J Clin Med. 2019 Mar 11;8(3):342. doi: 10.3390/jcm8030342 (PMC6463106; doi:10.3390/jcm8030342)
Supplement: Supplementary file 1 [file jcm-08-00342-s001.pdf]

Supplementary Material

Common inflammation-related candidate gene variants and acute kidney injury in 2647 critically ill Finnish patients

Laura M. Vilander, MD[1]; Suvi T. Vaara, MD, PhD[1]; Mari A. Kaunisto, PhD[2]; Ville Pettilä, MD, PhD[1]; the FINNAKI study group.

[1] Division of Intensive Care Medicine, Department of Anesthesiology, Intensive Care and Pain Medicine, University of Helsinki and Helsinki University Hospital, Helsinki, Finland
[2] Institute for Molecular Medicine Finland (FIMM), HiLIFE, University of Helsinki, Helsinki, Finland
* Correspondence: laura.vilander@helsinki.fi; Tel.: +358505702266

Table of contents

Exclusion criteria for the study **2**

Primer sequences **3**

Abbreviations for genes **4**

Percentages of imputed values in covariates **5**

Results for analyses 2 and 3 .... **6**

Haplotype analyses results for IL6 .... **10**

Retrospective power calculation for each variant **11**

Summary-level data **13**

Exclusion criteria for the study

We excluded patients with

1) with end-stage renal disease or maintenance dialysis,

2) re-admitted and who previously received renal replacement therapy (RRT),

3) with insufficient language skills or with no permanent residency in Finland,

4) transferred between study ICUs (if already in the study for 5 days),

5) admitted to intermediate care, and/or

6) organ donors.

Primer sequences

	SNP capture primers		Extension primer
rs1474347	ACGTTGGATGTGCAAGGAAGGTTTTTGGAG	ACGTTGGATGACTTGGGTTCAGTTCCAAGC	GTTCCAAGCTCACCT
rs3025039	ACGTTGGATGAGACTCCGGCGGAAGCATT	ACGTTGGATGCTCGGTGATTTAGCAGCAAG	GCGGGTGACCCAGCA
rs2493133	ACGTTGGATGTGAGCAATCCCCCCATCTAT	ACGTTGGATGGTGGCGCATAGTGATTTTTC	CCCCCATCTATCACCTC
rs1800796	ACGTTGGATGTGGAGACGCCTTGAAGTAAC	ACGTTGGATGGAGTTCTTCTGTGTTCTGGC	GCAGTTCTACAACAGCC
rs1050851	ACGTTGGATGCTGCAGGTTGTTCTGGAAGT	ACGTTGGATGAAAGGCACTGACCATGGAAG	tTGAAGGGAGACCTGGC
rs1800795	ACGTTGGATGAGCCTCAATGACGACCTAAG	ACGTTGGATGGATTGTGCAATGTGACGTCC	cGTGACGTCCTTTAGCAT
rs13306435	ACGTTGGATGCCCTCCACTGCAAAGGATTT	ACGTTGGATGTGGCATTTGTGGTTGGGTCA	ggtaAGGGGTGGTTATTGC
rs10421768	ACGTTGGATGACATCTCAAGGGTCTGACAC	ACGTTGGATGGAACACTAGATAGCCCTGAG	gcggGTGCCCGATCCGCACG
rs2243639	ACGTTGGATGCTCTTTCCACTGCTCACCTG	ACGTTGGATGTAAGGGAGAGCGAGGTGTC	caCGTGGAGTCCCTGGAAAC
rs2868371	ACGTTGGATGGCACAAGTTCATCTGCTTAG	ACGTTGGATGAATCTGGTGTGGTTGTTAGG	ACTGCCTGAATTCACATCTTT
rs2069842	ACGTTGGATGAGAAAAAGGTGGGTGTGTCC	ACGTTGGATGCATTGCATCCCTGAGTTGTC	cccccCATTCCCTCAACTTGGT
rs721917	ACGTTGGATGTCCACTGAGCTACACATGAC	ACGTTGGATGAGAAATGAAGACCTACTCCC	gggcCCTACTCCCACAGAACAA
rs2069830	ACGTTGGATGTTCGGTCCAGTTGCCTTCTC	ACGTTGGATGGCGGCTACATCTTTGGAATC	gCATCTTTGGAATCTTCTCCTG
rs10262995	ACGTTGGATGGAGAGAGTGTTGAATTGTGG	ACGTTGGATGTTTGTCCTATCAGGCCATCC	cccaACATCTCCCTCTAATCATG
rs4073	ACGTTGGATGTGTTCTAACACCTGCCACTC	ACGTTGGATGCTGAAGCTCCACAATTTGGT	TCCACAATTTGGTGAATTATCAA
rs10499563	ACGTTGGATGAAGCCTGGTCTGGCCTGTAT	ACGTTGGATGGATTCTAGAGCCCTGGTAAG	ATTTCTTAATTATTATACAAGCACA
rs7208693	ACGTTGGATGTCCATGGAGCTCAGCACCAA	ACGTTGGATGGCCCATTCCTGACTTTGTGA	CCACCTCCCCCAGGA
rs2010963	ACGTTGGATGAGAGAAGTCGAGGAAGAGAG	ACGTTGGATGTCCGGCGGTCACCCCCAAAA	TGCGAGCAGCGAAAG
rs4680	ACGTTGGATGTTTTCCAGGTCTGACAACGG	ACGTTGGATGATCGAGATCAACCCCGACTG	CACACCTTGTCCTTCA
rs2070744	ACGTTGGATGACCAGGGCATCAAGCTCTTC	ACGTTGGATGTGTCATTCAGTGACGCACGC	CAAGCTCTTCCCTGGC
rs699	ACGTTGGATGGATTGACAGGTTCATGCAGG	ACGTTGGATGAGGTGTTGAAAGCCAGGGTG	GACTGGCTGCTCCCTGA
rs1800896	ACGTTGGATGATTCCATGGAGGCTGGATAG	ACGTTGGATGGACAACACTACTAAGGCTTC	tgCCTATCCCTACTTCCCC
rs1800629	ACGTTGGATGGATTTGTGTGTAGGACCCTG	ACGTTGGATGGGTCCCCAAAAGAAATGGAG	cccctAGGCTGAACCCCGTCC
rs11549465	ACGTTGGATGCTTCCAGTTACGTTCCTTCG	ACGTTGGATGTTGAGGACTTGCGCTTTCAG	cttcCCTTCGATCAGTTGTCA
rs10748825	ACGTTGGATGTGCCCACATTTGAATCCCTG	ACGTTGGATGCATCTCAAGATCCCTAACTG	gaaCTGGAACTTGTGATATGC
rs1617640	ACGTTGGATGTGAGAGACCAGCTAGTCTTG	ACGTTGGATGGCTGGGATTTACAGCTAAGG	atcaTGCTCTGGGAATCTCACTC
rs876493	ACGTTGGATGATCCATCTCCCTTAGTGTCC	ACGTTGGATGTGCGGGAGCAAGTACGGAG	gagttTCCGGGCTGCAGACACAC

Abbreviations for genes

TNFA, tumor necrosis factor alpha

IL6, interleukin 6

CXCL8, interleukin 8

IL10, interleukin 10

NOS3, nitric oxide synthase 3

NFKB1A, nuclear factor of kappa light polypeptide gene enhancer in B-cells inhibitor, alpha

AGT, angiotensinogen

VEGFA, vascular endothelial growth factor A

EPO, erythropoietin

SUFU, suppressor of fused homolog

HIF1A, hypoxia-inducible factor 1-alpha

PNMT, phenylethanolamine N-methyltransferase

MPO, myeloperoxidase

COMT, catechol-O-methyltransferase

HSPB1, heat shock protein family B (small) member 1

SFTPD, surfactant protein D

HAMP, hepcidin antimicrobial peptide

BBS9, Bardet-Biedl syndrome 9

Percentages of imputed values in covariates

We imputed missing data with median for continuous variables and “none” for categorical variables.

- liver failure, imputed 37(1.3% of 2846)
- body mass index (BMI), imputed 22 (0.8% of 2846)
- use of nonsteroidal anti-inflammatory drugs (NSAID) as permanent medication, imputed 116 (4.1% of 2846)
- use of warfarin as permanent medication, imputed 40 (1.4% of 2846)
- use of contrast dye prior to ICU admission, imputed 17 (0.6% of 2846)
- use of colloids prior to ICU admission, imputed 175 (6.1% of 2846)
- use of albumin prior to ICU admission, imputed 65 (2.3% of 2846)
- minimum platelet count, imputed 243 (8.5% of 2846)
- simplified acute physiology score (SAPS) II without renal and age points), imputed 34 (1.2% of 2846)
- cardiac surgery status, imputed 0
- sepsis status, imputed 0

Results for analyses 2 and 3

Table 4. Adjusted analysis; association of genetic variants with acute kidney injury (AKI) KDIGO Stages 2 and 3 compared to Stage 0. **Patients with chronic kidney disease included**. Odds ratios (OR) and confidence intervals (95% CI) are reported for each copy of minor allele.

**AKI KDIGO 23 to 0 (covariates)**	**SNP**	**patients**	**minor allele**	**MAF (cases/ controls)**	**additive logistic OR**	**95% CI**	***p***
TNFA	rs1800629	2319	A	0.15/0.14	1.10	0.91-1.32	0.34
IL10	rs1800896	2318	G	0.44/0.46	0.90	0.79-1.03	0.14
IL6	rs10499563	2341	C	0.15/0.14	0.98	0.81-1.18	0.81
	rs1800796	2346	C	0.028/0.034	0.89	0.60-1.32	0.57
	rs1800795	2337	G	0.47/0.47	0.99	0.87-1.13	0.92
	rs1474347	2334	A	0.47/0.47	0.99	0.87-1.13	0.89
	rs13306435	2348	A	0.030/0.034	0.93	0.64-1.36	0.70
CXCL8	rs4073	2341	A	0.40/0.42	0.93	0.81-1.07	0.32
NOS3	rs2070744	2319	C	0.34/0.36	0.94	0.82-1.09	0.42
NFKB1A	rs1050851	2344	A	0.15/0.17	0.95	0.79-1.14	0.59
AGT	rs699	1120	C	0.43/0.42	1.00	0.83-1.21	0.99
	rs2493133	2345	C	0.41/0.42	0.95	0.83-1.09	0.43
VEGFA	rs2010963	2315	C	0.22/0.24	0.88	0.75-1.04	0.13
	rs3025039	2343	T	0.16/0.14	1.21	1.00-1.45	0.047
EPO	rs1617640	2318	G	0.45/0.44	1.05	0.92-1.20	0.49
SUFU	rs10748825	2319	G	0.37/0.37	1.03	0.90-1.19	0.64
HIF1A	rs11549465	2318	T	0.052/0.043	1.20	0.88-1.64	0.25
PNMT	rs876493	2318	C	0.37/0.38	0.95	0.83-1.09	0.48

MPO	rs7208693	2319	A	0.11/0.11	0.92	0.75-1.14	0.47
COMT	rs4680	2318	G	0.47/0.45	1.10	0.96-1.26	0.17
HSPB1	rs2868371	2341	G	0.19/0.21	0.90	0.76-1.06	0.21
SFTPD	rs2243639	2348	T	0.39/0.40	0.94	0.83-1.08	0.41
	rs721917	2342	G	0.39/0.39	1.02	0.89-1.17	0.76
HAMP	rs10421768	2348	G	0.22/0.25	0.81	0.69-0.95	0.0090
BBS9	rs10262995	2349	T	0.039/0.042	0.86	0.61-1.21	0.39

Covariates in the model: liver failure, body mass index (BMI), use of nonsteroidal anti–inflammatory drugs (NSAID) or warfarin as permanent medication, use of contrast dye prior to ICU admission, use of colloids prior to ICU admission, use of albumin prior to ICU admission, minimum platelet count, simplified acute physiology score (SAPS) II without renal and age points, sepsis, and cardiac surgery. Abbreviations: AKI, acute kidney injury; KDIGO, Kidney Disease: Improving Global Outcomes; SNP, single nucleotide polymorphism; MAF, minor allele frequency; OR, odds ratio; CI, confidence interval; TNFA, tumor necrosis factor alpha; IL10, interleukin 10; IL6, interleukin 6; CXCL8, interleukin 8; NOS3, nitric oxide synthase 3; NFKB1A, nuclear factor of kappa light polypeptide gene enhancer in B-cells inhibitor, alpha; AGT, angiotensinogen; VEGFA, vascular endothelial growth factor A; EPO, erythropoietin; SUFU, suppressor of fused homolog; HIF1A, hypoxia-inducible factor 1-alpha; PNMT, phenylethanolamine N-methyltransferase; MPO, myeloperoxidase; COMT, catechol-O-methyltransferase; HSPB1, heat shock protein family B (small) member 1; SFTPD, surfactant protein D; HAMP, hepcidin antimicrobial peptide; BBS9, Bardet-Biedl syndrome 9.

Table 5. Association of genetic variants with acute kidney injury (AKI) KDIGO **Stages 1, 2 and 3** compared to Stage 0. Odds ratios (OR) and confidence intervals (95% CI) are reported for each copy of minor allele.

	**SNP**	**patients**	**minor allele**	**MAF (cases/ controls)**	**additive logistic OR**	**95% CI**	***p***
TNFA	rs1800629	2601	A	0.14/0.14	1.01	0.87–1.18	0.87
IL10	rs1800896	2600	G	0.45/0.46	0.94	0.84–1.05	0.26
IL6	rs10499563	2632	C	0.15/0.14	1.05	0.91–1.23	0.50
	rs1800796	2637	C	0.031/0.034	0.92	0.67–1.26	0.61
	rs1800795	2629	G	0.47/0.47	0.98	0.88–1.10	0.75
	rs1474347	2626	A	0.47/0.47	0.98	0.88–1.10	0.75
	rs13306435	2638	A	0.031/0.033	0.94	0.68–1.28	0.68
CXCL8	rs4073	2632	A	0.40/0.42	0.94	0.85–1.07	0.43
NOS3	rs2070744	2600	C	0.34/0.36	0.92	0.81–1.03	0.13
NFKB1A	rs1050851	2636	A	0.15/0.17	0.93	0.80–1.08	0.32
AGT	rs699	1261	C	0.42/0.42	0.99	0.84–1.16	0.88
	rs2493133	2635	C	0.41/0.42	0.96	0.85–1.07	0.43
VEGFA	rs2010963	2597	C	0.22/0.24	0.92	0.80–1.05	0.19
	rs3025039	2633	T	0.16/0.14	1.17	1.01–1.37	0.041
EPO	rs1617640	2600	G	0.45/0.45	1.03	0.92–1.15	0.66
SUFU	rs10748825	2601	G	0.38/0.37	1.03	0.92–1.16	0.64
HIF1A	rs11549465	2600	T	0.047/0.042	1.11	0.85–1.45	0.44
PNMT	rs876493	2600	C	0.36/0.38	0.91	0.81–1.02	0.10
MPO	rs7208693	2601	A	0.11/0.12	0.91	0.77–1.09	0.32
COMT	rs4680	2599	G	0.47/0.46	1.04	0.93–1.16	0.48

HSPB1	rs2868371	2634	G	0.20/0.21	0.95	0.83–1.09	0.49
SFTPD	rs2243639	2638	T	0.40/0.40	0.98	0.88–1.10	0.78
	rs721917	2632	G	0.38/0.39	0.97	0.87–1.08	0.59
HAMP	rs10421768	2639	G	0.24/0.25	0.96	0.85–1.09	0.54
BBS9	rs10262995	2640	T	0.041/0.042	0.98	0.74–1.30	0.91

Abbreviations: AKI, acute kidney injury; KDIGO, Kidney Disease: Improving Global Outcomes; SNP, single nucleotide polymorphism; MAF, minor allele frequency; OR, odds ratio; CI, confidence interval; TNFA, tumor necrosis factor alpha; IL10, interleukin 10; IL6, interleukin 6; CXCL8, interleukin 8; NOS3, nitric oxide synthase 3; NFKB1A, nuclear factor of kappa light polypeptide gene enhancer in B-cells inhibitor, alpha; AGT, angiotensinogen; VEGFA, vascular endothelial growth factor A; EPO, erythropoietin; SUFU, suppressor of fused homolog; HIF1A, hypoxia-inducible factor 1-alpha; PNMT, phenylethanolamine N-methyltransferase; MPO, myeloperoxidase; COMT, catechol-O-methyltransferase; HSPB1, heat shock protein family B (small) member 1; SFTPD, surfactant protein D; HAMP, hepcidin antimicrobial peptide; BBS9, Bardet-Biedl syndrome 9.

Haplotype analyses results for *IL6*

Analysis 1, haploblock 1, rs10499563|rs1800796|rs1800795|rs1474347|rs13306435

**haplotype**	**OR**	***p***
TGGAA	0.91	0.63
TCGAT	0.89	0.56
CGGAT	1.03	0.73
TGGAT	1.01	0.88
TGCCT	0.98	0.78

Analysis 1, haploblock 2, rs1800795|rs1474347

**haplotype**	**OR**	***p***
CC	1.01	0.89
GA	0.99	0.86

Abbreviations: *IL6*, interleukin 6; OR, odds ratio.

Retrospective power calculation for each variant

Table 6. Retrospective power calculations for each studied variant. Acute kidney injury (AKI) KDIGO Stages 2 and 3 (N=625) compared to Stage 0 (N=1582). Genetic Power Calculator (http://zzz.bwh.harvard.edu/gpc/) was used for power calculations. Allele frequencies are from our study's controls (Stage 0), prevalence of the phenotype is 0.39 in our critically ill cohort, assumed genotype relative risk for heterozygotes 1.2 and for homozygotes 1.4, and the type I error rate was set to 0.005.

	**SNP**	**patients**	**MAF (control)**	**minor allele**	**Power (%)**
TNFA	rs1800629	2174	0.14	A	67.6
IL10	rs1800896	2173	0.46	G	90.8
IL6	rs10499563	2192	0.14	C	67.6
	rs1800796	2197	0.03	C	13.0
	rs1800795	2189	0.47	G	90.7
	rs1474347	2187	0.47	A	90.7
	rs13306435	2199	0.03	A	13.0
CXCL8	rs4073	2193	0.42	A	91.2
NOS3	rs2070744	2174	0.36	C	90.8
NFKB1A	rs1050851	2196	0.17	A	75.3
AGT	rs699	1047	0.42	C	91.2

	rs2493133	2196	0.42	C	91.2
VEGFA	rs2010963	2170	0.24	C	85.3
	rs3025039	2195	0.14	T	67.6
EPO	rs1617640	2173	0.45	G	90.9
SUFU	rs10748825	2174	0.37	G	91.0
HIF1A	rs11549465	2173	0.04	T	18.9
PNMT	rs876493	2173	0.38	C	91.1
MPO	rs7208693	2174	0.12	A	60.9
COMT	rs4680	2173	0.45	G	90.9
HSPB1	rs2868371	2194	0.21	G	82.0
SFTPD	rs2243639	2199	0.40	T	91.2
	rs721917	2193	0.39	G	91.1
HAMP	rs10421768	2199	0.25	G	86.2
BBS9	rs10262995	2200	0.04	T	18.9

Abbreviations: AKI, acute kidney injury; KDIGO, Kidney Disease: Improving Global Outcomes; SNP, single nucleotide polymorphism; MAF, minor allele frequency; OR, odds ratio; CI, confidence interval; TNFA, tumor necrosis factor alpha; IL10, interleukin 10; IL6, interleukin 6; CXCL8, interleukin 8; NOS3, nitric oxide synthase 3; NFKB1A, nuclear factor of kappa light polypeptide gene enhancer in B-cells inhibitor, alpha; AGT, angiotensinogen; VEGFA, vascular endothelial growth factor A; EPO, erythropoietin; SUFU, suppressor of fused homolog; HIF1A, hypoxia-inducible factor 1-alpha; PNMT, phenylethanolamine N-methyltransferase; MPO, myeloperoxidase; COMT, catechol-O-methyltransferase; HSPB1, heat shock protein family B (small) member 1; SFTPD, surfactant protein D; HAMP, hepcidin antimicrobial peptide; BBS9, Bardet-Biedl syndrome 9.

Summary-level data

Genotype frequencies			No AKI			AKI KDIGO Stage 1			AKI KDIGO Stage 2			AKI KDIGO Stage 3		
Variant	minor allele (b)	major allele (B)	bb	Bb	BB	bb	Bb	BB	bb	Bb	BB	bb	Bb	BB
RS1800896	G	A	22.2%	48.4%	29.4%	21.3%	48.0%	30.7%	17.1%	53.4%	29.5%	20.3%	48.2%	31.6%
RS699	C	T	17.4%	49.3%	33.2%	16.5%	47.0%	36.4%	21.7%	45.7%	32.6%	17.4%	48.2%	34.4%
RS1800629	A	G	2.4%	23.6%	74.0%	1.3%	25.1%	73.7%	3.0%	19.2%	77.8%	3.0%	26.4%	70.6%
RS2010963	C	G	6.3%	35.9%	57.8%	4.6%	35.4%	60.0%	3.9%	37.8%	58.4%	6.0%	31.8%	62.3%
RS1617640	G	T	20.3%	48.3%	31.4%	21.1%	50.3%	28.6%	21.5%	48.5%	30.0%	19.4%	49.6%	31.1%
RS2070744	C	T	13.3%	44.8%	41.9%	10.5%	45.4%	44.1%	12.0%	43.2%	44.9%	11.1%	44.9%	44.0%
RS10748825	G	A	12.9%	48.1%	39.0%	12.6%	49.7%	37.7%	13.7%	48.3%	38.0%	13.6%	47.2%	39.1%
RS11549465	T	C	0.2%	8.1%	91.6%	0.2%	8.6%	91.2%	0.9%	11.1%	88.0%	0.0%	9.1%	90.9%
RS876493	C	T	13.9%	47.6%	38.5%	12.0%	43.8%	44.2%	11.5%	41.9%	46.6%	15.5%	46.4%	38.1%
RS7208693	A	C	1.4%	20.1%	78.5%	1.1%	20.4%	78.5%	0.4%	19.2%	80.3%	2.1%	17.2%	80.6%
RS4680	G	A	21.0%	49.1%	30.0%	22.4%	47.0%	30.6%	21.8%	50.0%	28.2%	23.0%	48.9%	28.1%
RS2493133	C	T	17.4%	49.4%	33.2%	18.9%	45.8%	35.3%	16.9%	46.2%	36.9%	17.0%	47.9%	35.1%
RS4073	A	T	17.1%	49.0%	33.9%	15.2%	50.1%	34.7%	15.7%	44.5%	39.8%	18.4%	46.6%	35.0%
RS3025039	T	C	2.1%	24.1%	73.8%	2.1%	27.4%	70.6%	3.8%	20.7%	75.5%	2.7%	27.9%	69.3%
RS10499563	C	T	2.9%	23.0%	74.0%	1.4%	26.2%	72.3%	3.8%	19.5%	76.7%	2.1%	26.3%	71.6%
RS1800796	C	G	0.1%	6.5%	93.4%	0.2%	5.7%	94.1%	0.0%	3.8%	96.2%	0.0%	6.5%	93.5%
RS1800795	G	C	24.0%	46.3%	29.7%	20.7%	50.0%	29.3%	21.5%	49.8%	28.7%	21.2%	52.2%	26.6%
RS1474347	A	C	23.9%	46.3%	29.7%	20.5%	50.1%	29.4%	21.2%	50.0%	28.8%	21.1%	52.2%	26.7%
RS13306435	A	T	0.1%	6.6%	93.3%	0.0%	6.4%	93.6%	0.0%	6.3%	93.7%	0.2%	5.5%	94.3%
RS10262995	T	C	0.1%	8.1%	91.8%	0.2%	7.4%	92.4%	0.4%	7.1%	92.4%	0.0%	7.8%	92.2%
RS2868371	G	C	4.4%	33.0%	62.6%	4.9%	34.0%	61.1%	3.8%	32.1%	64.1%	4.0%	30.2%	65.8%
RS2243639	T	C	16.2%	48.0%	35.8%	16.8%	48.0%	35.1%	18.1%	37.1%	44.7%	17.2%	46.0%	36.8%
RS721917	G	A	15.5%	47.6%	36.9%	14.8%	44.6%	40.7%	17.7%	43.5%	38.8%	17.5%	44.0%	38.5%
RS1050851	A	G	3.1%	27.0%	69.9%	1.8%	26.8%	71.3%	2.5%	24.4%	73.1%	2.7%	25.3%	72.0%
RS10421768	G	A	6.1%	37.2%	56.8%	9.4%	33.4%	57.2%	5.9%	35.0%	59.1%	4.2%	34.0%	61.8%
